# A novel method for efficient closure of large mucosal defects using nylon loops combined with titanium clips after endoscopic submucosal dissection

**DOI:** 10.1055/a-2106-2383

**Published:** 2023-06-27

**Authors:** Geng Qin, Qian-qian Wang, Chang Tan

**Affiliations:** 1 Department of Gastroenterology, China-Japan Friendship Hospital, Beijing, China; 2 Peking university China-Japan Friendship School of Clinical Medicine, Beijing, China

Endoscopic submucosal dissection (ESD) is an effective treatment for early-stage gastrointestinal cancer 1 2 . However, difficult site lesions (e. g., colon flexion, right-side colon) and large mucosal defects (greater than 5 cm) result in post-ESD defect closures as an ongoing challenge in clinical practice 3 . Therefore, we report a novel method for the efficient closure of large mucosal defects after ESD using nylon loops combined with titanium clips.

We demonstrate the application of this method in a real case of post-ESD large defect closure ( Video 1 ). An 80-year-old man was found to have a circumferential 1/3 perineal lateral developmental tumor in the left posterior wall of the rectum 4–7 cm from the anal orifice with a pathology of high-grade intraepithelial neoplasia. The operation procedure was as follows. After complete debridement of the lesion by ESD ( Fig. 1 ), one titanium clip with a nylon loop was fixed at the midpoint of one side of the incision margin ( Fig. 2 ). Another titanium clip was fixed at the midpoint of the opposite side of the incision margin ( Fig. 3 ), and then the nylon loop was gathered to transform the oval-shaped defect into a “figure-eight-shaped” defect ( Fig. 4 ). The remaining defect was closed with an appropriate amount of additional titanium clips.

**Video 1** The application of nylon loop combined with titanium clips in a real case of large mucosal defect closure after endoscopic submucosal dissection.


**Fig. 1:**
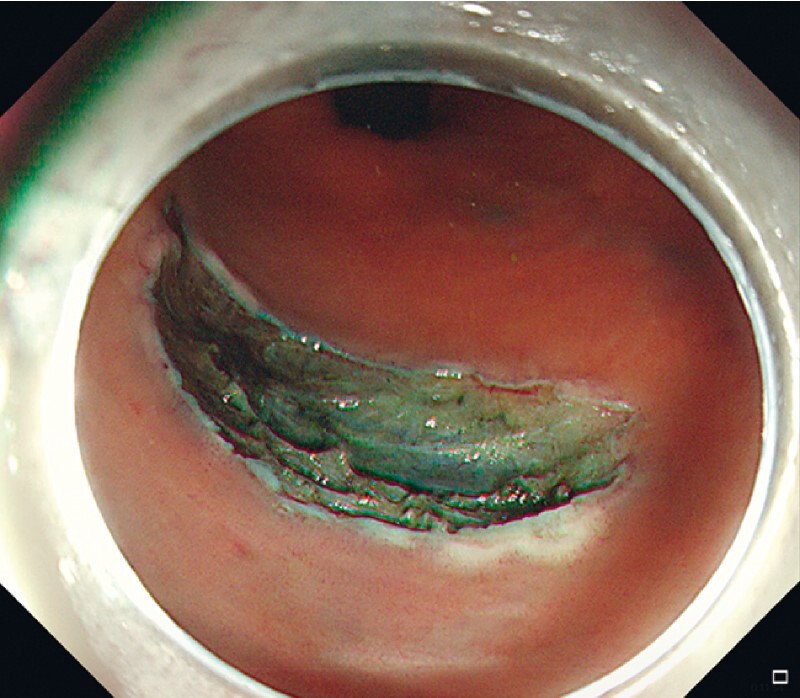
Complete dissection of the lesion, leaving a large mucosal defect.

**Fig. 2:**
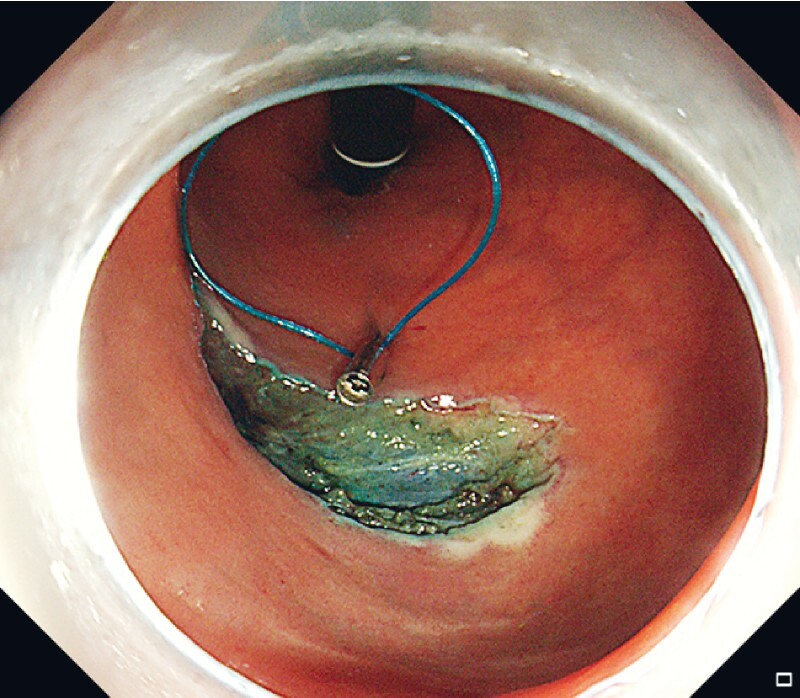
One titanium clip with a nylon loop was fixed at the midpoint of one side of the incision margin.

**Fig. 3:**
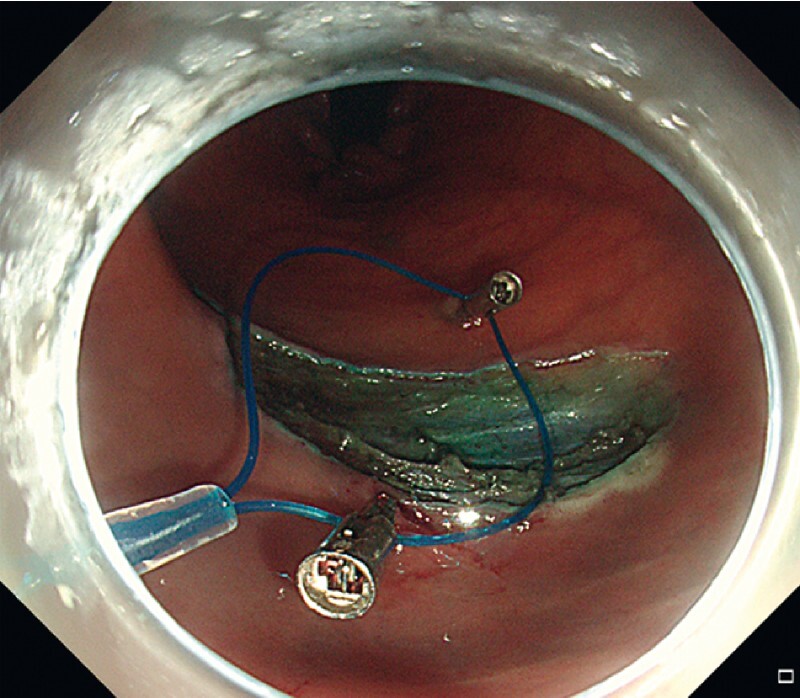
Another titanium clip with the nylon loop was fixed at the midpoint of the opposite side of the incision margin.

**Fig. 4:**
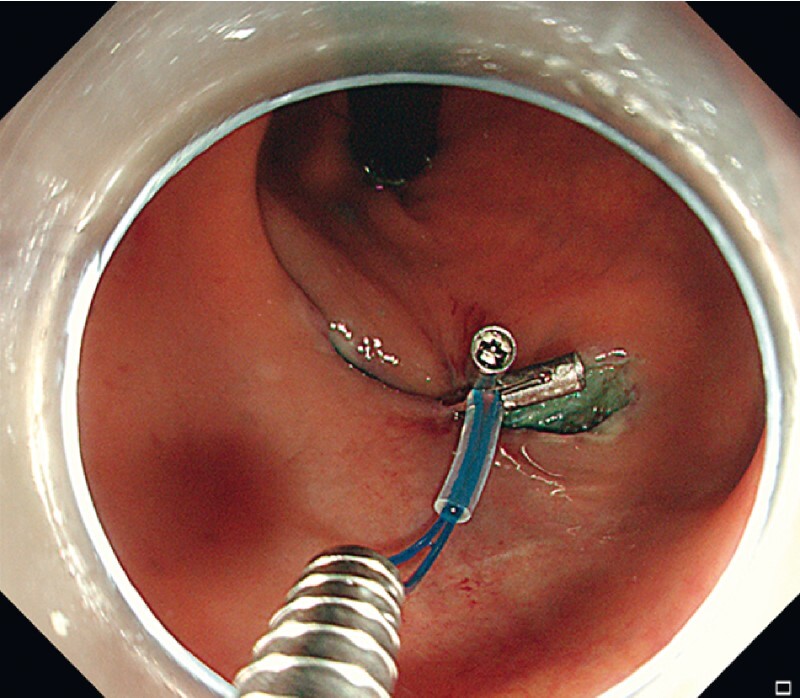
The nylon loop was gathered, and the oval-shaped defect was transformed into a “figure-eight-shaped” defect.

This method was applied to 50 patients with lesions ranging from 1/3–1/2 of the circumference of the bowel, and the mean closure time was 12.6 minutes. The mean number of titanium clips was 7.4, saving endoscopic closure time and the number of titanium clips compared to previous studies 4 . In addition, based on this, we have further developed a combination device consisting of a titanium clip and multiple nylon rings ( Fig. 5 ), for which a patent application has been submitted in China. The device can further reduce the endoscopic closure time to 7 minutes, significantly improving the efficiency of endoscopic closure of large mucosal defects.

**Fig. 5:**
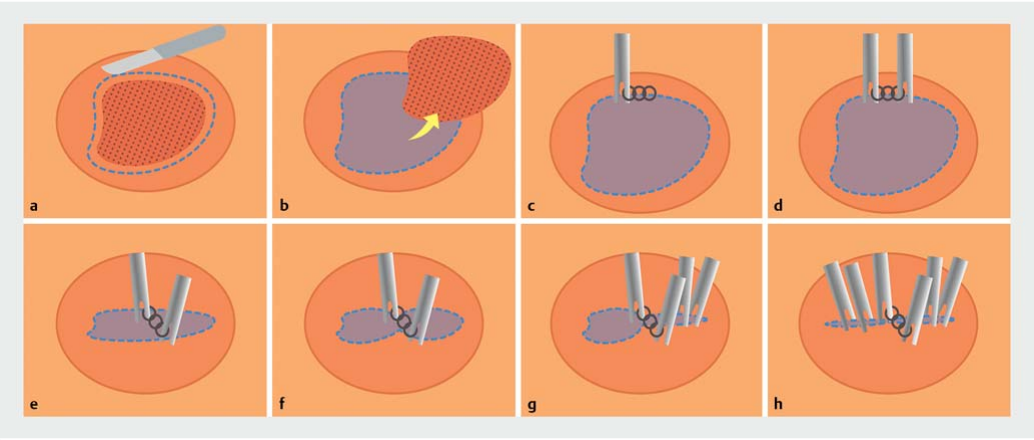
Schematic diagram for efficient closure of large mucosal defects using a combination device of a titanium clip combined with multiple nylon rings after endoscopic submucosal dissection (ESD). **a** ESD was performed to remove early-stage gastrointestinal cancer. **b** Complete dissection of the lesion, leaving a large mucosal defect. **c** One titanium clip with multiple nylon rings was fixed at the midpoint of one side of the incision margin. **d** Another titanium clip was fixed at the midpoint of the opposite side of the incision margin. **e** The number of nylon rings between two titanium clips is determined by the size of the mucosal defect. **f** The oval-shaped defect was transformed into a “figure-eight-shaped” defect. **g–h** Additional titanium clips were added to the remaining defect until it was completely closed.

Endoscopy_UCTN_Code_TTT_1AQ_2AD

## Notes

Endoscopy E-Videos

https://eref.thieme.de/e-videos
*E-Videos* is an open access online section of the journal *Endoscopy* , reporting on interesting cases and new techniques in gastroenterological endoscopy. All papers include a high-quality video and are published with a Creative Commons CC-BY license. Endoscopy E-Videos qualify for HINARI discounts and waivers and eligibility is automatically checked during the submission process. We grant 100% waivers to articles whose corresponding authors are based in Group A countries and 50% waivers to those who are based in Group B countries as classified by Research4Life (see: https://www.research4life.org/access/eligibility/ ). This section has its own submission website at https://mc.manuscriptcentral.com/e-videos
